# Comparative dosimetry of radiography device, MSCT device and two CBCT devices in the elbow region

**DOI:** 10.1002/acm2.13245

**Published:** 2021-04-03

**Authors:** Juha Koivisto, Maureen van Eijnatten, John Ludlow, Timo Kiljunen, Xie‐Qi Shi, Jan Wolff

**Affiliations:** ^1^ Department of Physics University of Helsinki Helsinki Finland; ^2^ Department of Oral and Maxillofacial Surgery/Oral Pathology VU University Medical Center Amsterdam The Netherlands; ^3^ University of North Carolina Adams School of Dentistry Chapel Hill NC USA; ^4^ International Docrates Cancer Center Helsinki Finland; ^5^ Section of Oral Maxillofacial Radiology Department of Clinical Dentistry Faculty of Medicine and Dentistry University of Bergen Bergen Norway; ^6^ Department of Oral Maxillofacial Surgery University Medical Center Hamburg‐Eppendorf Hamburg Germany; ^7^ Division of Regenerative Orofacial Medicine Hamburg Germany

**Keywords:** CBCT, effective dose, elbow, x‐ray radiation

## Abstract

The aim of the study was to estimate and to compare effective doses in the elbow region resulting from four different x‐ray imaging modalities. Absorbed organ doses were measured using 11 metal oxide field effect transistor (MOSFET) dosimeters that were placed in a custom‐made anthropomorphic elbow RANDO phantom. Examinations were performed using Shimadzu FH‐21 HR radiography device, Siemens Sensation Open 24‐slice MSCT‐device, NewTom 5G CBCT device, and Planmed Verity CBCT device, and the effective doses were calculated according to ICRP 103 recommendations. The effective dose for the conventional radiographic device was 1.5 µSv. The effective dose for the NewTom 5G CBCT ranged between 2.0 and 6.7 µSv, for the Planmed Verity CBCT device 2.6 µSv and for the Siemens Sensation MSCT device 37.4 µSv. Compared with conventional 2D radiography, this study demonstrated a 1.4–4.6 fold increase in effective dose for CBCT and 25‐fold dose for standard MSCT protocols. When compared with 3D CBCT protocols, the study showed a 6‐19 fold increase in effective dose using a standard MSCT protocol. CBCT devices offer a feasible low‐dose alternative for elbow 3D imaging when compared to MSCT.

## 1 INTRODUCTION

A fall onto an outstretched arm is a typical cause for radial head fractures.^1^, ^2^ Such traumatic injuries are particularly common in children and adults who engage in sports activities.^3^, ^4^ To date, conventional radiographs play a major role in primary elbow diagnostics comprising anterior‐posterior (AP) and lateral (LAT) projections.^5^ However, it must be noted that complex fractures are often difficult to visualize using two‐dimensional (2D) radiographic images where skeletal objects may become superimposed by adjacent bony structures.^6^ In such cases, multi slice radiographs need to be acquired using computed tomography (CT) to provide highly detailed 3D information on occult and other complex fractures.^7^ Furthermore, multi‐slice computed tomography devices (MSCT) provide all the necessary diagnostic information that previously required two or more 2D projections.^8^ However, a major drawback resulting from MSCT examinations is that they are often associated with a significantly higher radiation dose than conventional 2D transmission radiography.^9^ Another challenge faced when using MSCT for elbow examinations is the additional radiation dose to other body parts. In order to minimize excessive radiation dose, the internal organs of the body the arm should not be positioned adjacent to the body. Respectively, if the arm is extended upward, a part of the head may become irradiated by the primary or scattered radiation increasing the effective dose to the brain and to the bone marrow dose in the calvaria.^10^, ^11^

In recent years, cone beam computed tomography (CBCT) technology has found a new application for imaging extremities,^12^ thus offering a versatile alternative to MSCT devices. In clinical settings, CBCT images are particularly useful in detecting subtle and non‐displaced radial head fractures and for staging of intra‐articular fractures with entrapment of fragments within the elbow joint.^13^ The key benefits of CBCT technology are that they perform high‐resolution imaging of the extremities using less time than radiographs and multi‐detector computed tomography (MDCT) and lower radiation dose than MDCT.^14^, ^15^, ^16^ Furthermore, CBCT images of extremities (arms/legs) can be undertaken without irradiating other body parts.

Regardless of the x‐ray examination modality, radiation dose is always present, and the diagnostic benefits of all examinations should be weighed against the radiation risk that they induce. To the best of the authors’ knowledge, there are currently no contemporaneous studies involving the x‐ray radiation risk in the elbow region. Therefore, the aims of this study were to assess and compare the organ and effective doses in the elbow resulting from conventional radiography, MSCT, and two CBCT devices using manufacturer‐recommended elbow protocols.

## 2 MATERIALS AND METHODS

### 2.A X‐ray devices

Absorbed organ doses were measured using a conventional high‐resolution direct digital radiographic device (model FH‐21 Shimadzu Corporation, Kyoto, Japan), a 24‐slice MSCT device (model Sensation, Siemens, Forchheim, Germany), and two CBCT devices: (model NewTom 5G^®^, QR, Verona, Italy and model Verity, Planmed Oy, Helsinki, Finland).

In order to perform effective and absorbed dose comparisons, the field of view (FOV) of the conventional radiographic device and the MSCT scanner were chosen to match the FOV (13 cm × 16 cm) of the Planmed Verity CBCT scanner. The Planmed Verity radiation doses were assessed using the novel “Ultra Low Dose” (ULD) protocol, the NewTom 5G “Standard Scan” setting radiation dose was measured using three different FOVs (12 cm × 8 cm, 15 cm × 12 cm, 18 cm × 16 cm), and for the “HiRes” setting, the dose was measured using a 12 cm × 8 cm FOV. Since the diagnosis of elbow fractures is commonly based on AP and LAT projections, the sum of the doses obtained using the 2D projections served as a benchmark for the dose comparisons.

The exposure settings used in this study were based on a publication by Huang et al.^14^ In their study, Huang et al. compared effective dose values with corresponding image quality in the elbow region using a CBCT and MSCT device (Planmed Verity CBCT: (75–96 kVp, 3.8–12 mA, Philips and GE MDCT devices: 120 kVp, 200 mAs). (Table 1). The NewTom 5G device, however, uses a fixed 110 kVp tube voltage and automatically adjusted mA value based on two scout images of the region of interest (Table 1). The source‐to‐detector distance remained unchanged during the scanning procedure. However, the cone beam angle of the NewTom 5G scanner varied between 10.3 (small FOV) and 15.4 degrees (large FOV) between the different FOVs. (Table 5.)

**Table 1:** Exposure parameters of conventional radiography device, CBCT and MSCT scanners.

	Shimadzu	Siemens	NewTom 5G				Planmed
	FH‐21 HR	Sensation	CBCT				Verity
	AP+LAT	Open	"Hi Res"	"Standard Scan"			CBCT
	Radiography	MSCT	12 × 8	12 × 8	15 × 12	18 × 16	13 × 16
Potential (kV)	59	120	110	110	110	110	92
Tube current (mA)^a^	—	115	5.13	0.97	0.72	0.66	5
Exposure time (s)	—	1	5.4	3.6	3.6	3.6	4.5
Q (mA*s)	12.6	115	27.7	3.5	2.6	2.4	22.5
CTDIvol (mGy)	—	11.0	4.40	1.16	1.15	1.14	—
DLP (mGy *cm)	—	179	39.6	10.5	14.7	18.2	—
Filtration (mm Al eq.)	1.5	6.8	1.4	1.4	1.4	1.4	8.1
Focal size (mm)	1.2	0.7	0.3	0.6	0.6	0.6	0.5
Anode angle (º)	12º	9º	15º	15º	15º	15º	5º
Slice thickness (mm)	—	2	—	—	—	—	—
Pitch (mm)	—	0.5	—	—	—	—	—
Voxel H (mm)	—	—	0.15	0.3	0.3	0.3	0.4
Voxel L (mm)	—	—	0.15	0.3	0.3	0.3	0.4
Voxel W (mm)	—	—	0.15	0.3	0.3	0.3	0.4
Scan angle (º)	—	360º	360º	360º	360º	360º	210º
Frame number	—	—	360	360	360	360	300
Scan height (mm)	130	130	80	80	120	160	130
Scan diam. (mm)	160	160	120	120	150	180	160

^a^ For NewTom 5G CBCT: mA is automatically adjusted by "SafeBeam™" acquisition technique.

### 2.B Phantom

**Fig. 1:**
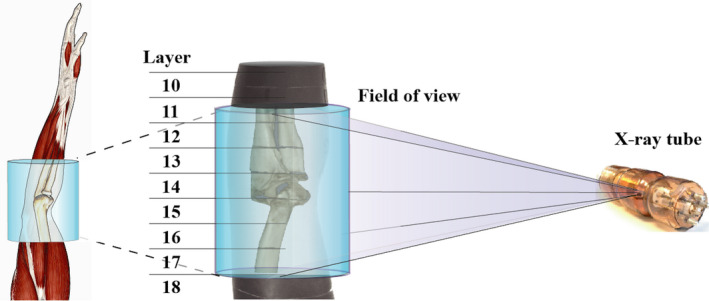
A schematic illustration of an anthropomorphic arm phantom with the exposed field of view.

All organ radiation dose measurements were performed using a specifically designed anthropomorphic RANDO adult arm phantom (Radiation Analogue Dosimetry System; The Phantom Laboratory, Salem, NY, USA). The phantom contains human bones that were encased within a soft tissue equivalent material to make the phantom match the radiation scattering and attenuation properties of the human elbow. The full arm phantom was sliced into 24 detachable layers that were numbered from 1 to 24 from the tip of the fingers to the upper arm (Fig. 1).

**Fig. 2:**
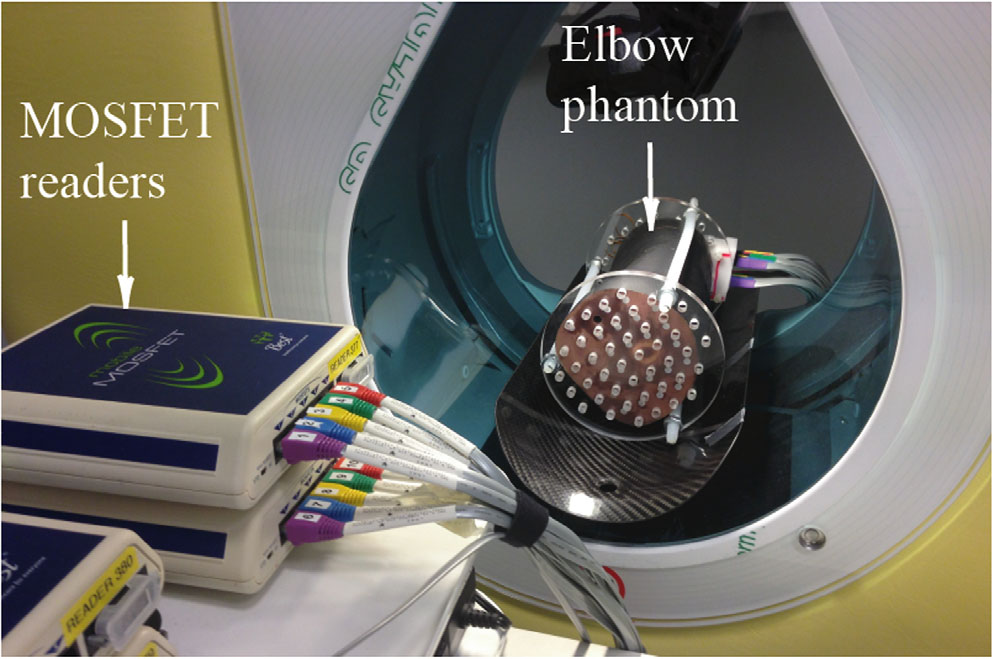
Typical setup of an anthropomorphic elbow phantom placed into the bore of a CBCT device and the MOSFET readers.

The layers were 25‐mm thick and had a 1.5 cm × 1.5 cm grid of 5‐mm diameter holes for the placement of MOSFET dosimeters. Each dosimeter void was factory fitted with a soft tissue equivalent plug that allowed the correct positioning of 11 MOSFET dosimeters. Figure 2 shows a typical setup of an anthropomorphic elbow phantom loaded with MOSFET dosimeters that is positioned into the bore of a CBCT device.

### 2.C Dosimeters

#### 2.C.1 Reference dosimeter

Before putting the MOSFET dosimeters into use, they were calibrated using a RADCAL 1015 dosimeter equipped with RADCAL 10X5‐6 ionization chamber (Radcal Corporation, Monrovia, CA, USA). The calibration chain is traceable to primary dosimetry laboratory (PSDL) via the secondary dosimetry laboratory (SSDL) at the Finnish Radiation and Nuclear Safety Laboratory. Prior to making the measurements, all 11 MOSFET dosimeters were calibrated for photon energy response, dose dependency, uncertainty, and angular sensitivity divergences according to the protocols presented in previous studies by Koivisto et al.^16^, ^17^, ^18^

#### 2.C.2 MOSFET dosimeters

The absorbed radiation doses required for the effective dose calculations were measured using a mobile TN‐RD‐70‐W20 MOSFET device. The measurement system comprises 11 reinforced high‐sensitivity TN‐1002RD‐H dosimeters, three TN‐RD‐16 reader modules, and TN‐RD‐75 M software (Best Medical, Ottawa, ON, Canada). The MOSFET device uses a TN‐RD‐38 wireless Bluetooth transceiver to transfer the data to a computer where the MOSFET dosimeter initializations and dose registrations are performed. The MOSFET dosimeter sensitivity can be selected using a switch on the reader device for high or low bias voltage that subsequently provides high or low sensitivity, respectively. In this study, the high sensitivity setting was applied to attain the best accuracy and to minimize dose related inaccuracies.

**Fig. 3:**
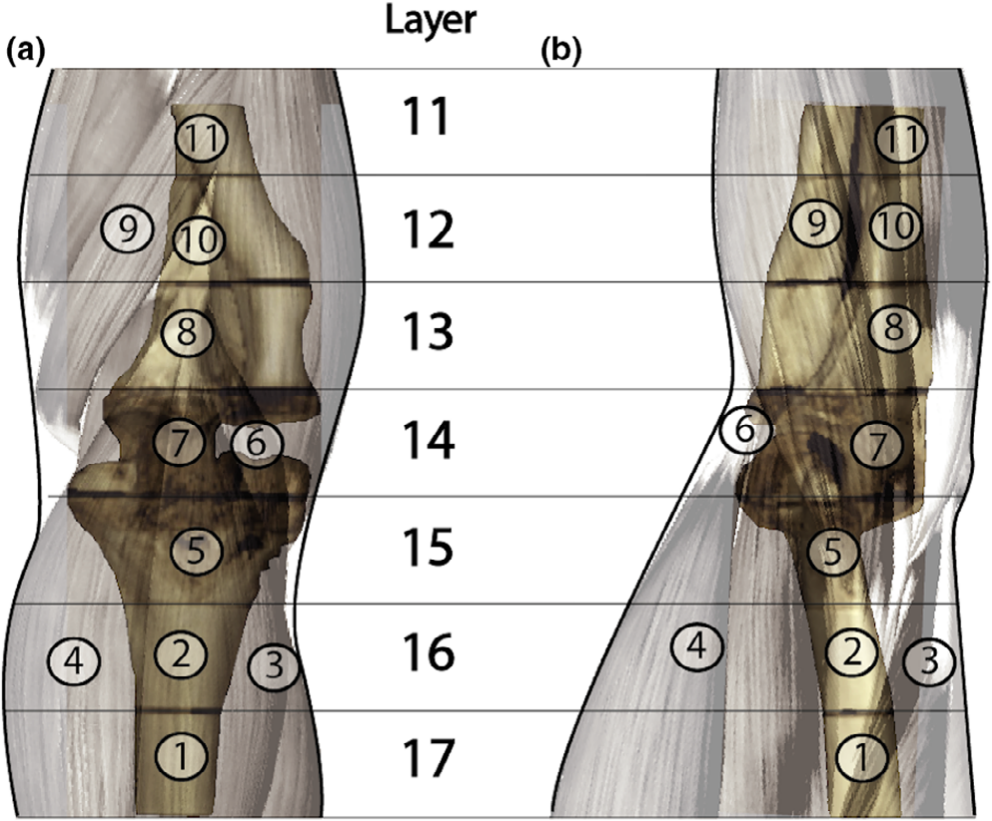
The placement of MOSFET dosimeters in the elbow phantom [AP (a), LAT (b)].

All 11 MOSFET dosimeters were positioned in predrilled voids located in the layers (11 to 17) of the phantom that covered the most radiosensitive organs (Table 2, Fig. 3).^19^ The highest contributor to the effective dose in extremities is bone marrow.^16^, ^20^, ^21^ Therefore, one dosimeter was placed within the bone (humerus, radius) in each layer to attain accurate dose detection and to provide a means to evaluate the dose uniformity amongst the different x‐ray modalities. The remaining dosimeters were used to measure the skin, bone surface, muscle and lymphatic (cubital) nodes.

**Table 2:** MOSFET dosimeter locations in anthropomorphic RANDO elbow phantom.

Dosimeter no	Layer	Location	Tissue
1	17	Humerus	Bone Marrow/Bone surface
2	16	Humerus	Bone Marrow/Bone surface
3	16	Triceps brachii	Bone surface/Muscle
4	16	Biceps brachii	Skin/Muscle
5	15	Humerus	Bone marrow/Bone surface
6	14	Cubital nodes/	Bone marrow/Bone surface/Lymphatic nodes
7	14	Ulna	Bone marrow
8	13	Ulna	Bone marrow/Bone surface
9	12	Radius/Flexor carpi ulnaris	Bone surface/Muscle/Skin
10	12	Ulna	Bone marrow
11	11	Ulna	Bone marrow/Bone surface

### 2.D Equivalent dose

The equivalent or radiation weighted dose *H_T_* of the irradiated tissues *T* were calculated using the following equation^22^:

(1) $$H_{T}=w_{R}\sum_{i}fi\cdot D_{\mathrm{Ti}}$$

In this equation, w_R_ is the radiation weighting factor (w_R_ = 1 Sv/Gy for x‐rays), *f_i_* is the mass fraction of tissue *T* in phantom layer *i*, and D*_Ti_* is the average absorbed dose of tissue *T* in layer *i*. The summation was performed over all exposed layers (11 to 17). In this study, the mass fraction (*f_i_*) denotes the coverage of each tissue in relative scale compared with the total tissue mass in the corresponding organ in the body (Table 3.) The studied organs were exposed by the incident and scattered radiation during the examinations. The dosimetric assessment was, however, performed using similar coverage regardless of the positioning of the beam to the phantom layers.^16^

**Table 3:** ICRP 103 weighting factors (*w_T_*), fractions irradiated *(f_i_*), and and dosimeters used to calculate effective dose.

Organ	w_T_	f_i_	Dosim. No.
Bone marrow	0.12		
Ulna		0.19%	7, 8, 10, 11
Radius		0.21%	6, 8, 10, 11
Humerus		0.60%	1, 2, 5, 6
Bone surface	0.01		
Ulna		0.40%	6, 9
Radius		0.39%	6, 9
Humerus		0.93%	1, 2, 5, 6
Skin	0.01		
Flexor carpi ulnaris		0.83%	9
Extensor carpi radialis longus		0.80%	8
Bicep		0.81%	4
Tricep		0.42%	3
Remainder	0.12		
Lymphatic nodes			
Cubital nodes		5.00%	6
Muscle			
Flexor carpi ulnaris		0.04%	9
Extensor carpi radialis longus		0.05%	6
Brachioradialis		0.18%	6
Bicep		0.22%	4
Tricep		0.40%	3

### 2.E Tissue mass factions

#### 2.E.1 Bone marrow

Bone marrow (BM) is one of the largest and most radiosensitive tissues in the human body. According to a study by Hindorf et al.,^23^ the total bone marrow content represents 4% of the total body weight. However, the active radiosensitive red bone marrow (RBM) comprises only one‐third of the total bone marrow weight.^24^ In the present study, the bone marrow volume (cm^3^) was assessed by measuring the cross section of all bones embedded in the phantom and multiplying them by the corresponding layer thickness (2.5 cm). The bone marrow volumes were summed for layers 10 to 17 to obtain the total bone marrow volume. The RBM weight was subsequently calculated as one‐third of the BM volume multiplied by the density of red bone marrow.^25^ Since bone marrow is one of the most radiosensitive organs, the RBM mass was also calculated based on data provided by Iwata et al.^26^ and using the RBM distribution according to a recent publication by Machann et al.^27^ According to both assessment methods the bone marrow in the elbow region represents 1.0% of the total bone marrow quantity in the human body.

#### 2.E.2 Bone surface

The bone surface in the elbow area was calculated using the bone surface‐to‐volume ratio according to ICRP Publication 70,^25^ the percentage of total fresh bone weights defined in ICRP Publication 89^24^ and the irradiated fractions of ulna, radius, and humerus based on the bone lengths according to a study by Schlenker et al.^28^ The bone surface in the exposed region was estimated to represent 1.7% of the total body bone surface.

#### 2.E.3 Skin

The skin area was calculated in layers 11 to 17. Each layer perimeter was multiplied by the 2.5‐cm phantom layer thickness and summed to attain the total skin area. The skin fraction in the elbow region was estimated by dividing the resulting value by the total skin area calculated using the Du Bois and Du Bois equation.^29^ The fraction of skin in the exposed volume was equivalent to 2.9% of the whole‐body skin.

#### 2.E.4 Remainder tissues

The effective dose contributions of the remainder tissues were calculated according to ICRP Publication 103 recommendations.^19^ The elbow region contains only two remainder organs, namely muscles and lymphatic nodes, also known as cubital nodes.

#### 2.E.5 Muscles

The total muscle mass was assessed by summing the fraction of each muscle in the elbow region according to a study by Holzbaur et al.^30^ The muscle mass fractions of each muscle were summed for layers between 11 and 17. The total body muscle mass in the elbow was calculated by dividing the result by the total 28 000 g body muscle mass.^31^, ^32^ It was estimated that muscles in the exposed region represent 0.9% of the total body muscle mass.

#### 2.E.6 Lymphatic nodes

The lymphatic node content in the elbow was evaluated using data published by Luscieti et al.^33^ and the results of a whole‐body lymphoscintigraphy examination performed at the Docrates Cancer Center in Helsinki, Finland.^16^ According to the study by Luscieti et al., the adult cubital node cross section in the elbow region was comparable with the popliteal fossa cross section located in the knee area. Furthermore, based on the lymphoscintigraphy, it was estimated that the popliteal fossa in the knee area contains 5% of the total lymphatic nodes. Since the lymphatic node content (cubital nodes) in the elbow and knee were comparable, the same (5%) lymphatic node mass fraction was used.

### 2.F Effective dose

The effective dose calculation was performed according to the International Commission of Radiological Protection (ICRP) guidelines.^19^ Although the effective dose is not a physical quantity, it provides a useful means to assess the stochastic risk between different imaging techniques and protocols for unevenly distributed organ doses in the body.^21^ The effective dose (E) was calculated from measured absorbed organ doses using the following equation:

(2) $$E=\sum_{T}w_{T}\cdot H_{T},$$

where *w_T_* is the weighting factor of tissue (*T*) and *H_T_* is the equivalent dose in tissue (*T*). All effective dose contributions were calculated using their specific fractions irradiated, weighting factors and adult organ dose compositions).^19^, ^33^ Furthermore, given that it may be difficult to compare the effective doses resulting from radiography, CBCT and MSCT devices due to the different exposure settings, their current — exposure time — product (mA * s) normalized effective doses (µSv/mAs) were calculated (Table 4). The tissue mass fractions (*f_i_*) and the ICRP Publication 103 weighting factors *w_T_* used in the calculations are presented in Table 3.

**Table 4:** Equivalent and effective doses (µSv) for the imaging of elbow with MSCT, CBCT, and radiography devices.

Tissue	Shimadzu	Siemens	NewTom 5G				Planmed
	FH‐21 HR	Sensation	CBCT				Verity
	AP+LAT	Open	"Hi Res"	"Standard Scan"			CBCT
	Radiography	MSCT	12 × 8	12 × 8	15 × 12	"18 × 16"	13 × 16
Bone marrow	0.6	19.0	3.4	1.0	1.2	1.0	1.3
Bone surface	0.1	2.7	0.4	0.1	0.2	0.1	0.2
Skin	0.2	5.5	0.8	0.2	0.3	0.3	0.4
Remainder							
Lymphatic nodes	0.4	8.5	2.0	0.6	0.7	0.5	0.6
Muscle	0.1	1.6	0.2	0.1	0.1	0.1	0.1
Effective dose	1.5	37.4	6.7	2.0	2.5	2.1	2.6
Norm.E (µSv/mAs)^a^	0.12	0.32	0.24	0.58	0.95	0.86	0.11
Effective dose ratio^b^	1.0	25.4	4.6	1.4	1.7	1.4	1.8

^a^ Exposure unit (mAs) normalized effective dose.

^b^ Dose compared to the radiographic device.

### 2.G Uncertainty analysis

The characterization involved the uncertainty of 11 individual dosimeters that were accounted for in this study. The absorbed dose uncertainty of a MOSFET dosimeter depends on the amount of dose exposure^18^ and due to the statistical nature, on the number of samples that were used for averaging. The type A standard absorbed dose (1SD) uncertainty (u_A_) was calculated from the 11 dosimeter uncertainties using quadratic summation of the average value of 10 repeated measurements according to a previous study by Koivisto et al.^16^ The combined type B uncertainty was calculated from the quadratic summation of all estimated uncertainties.

## 3 RESULTS

### 3.A Conventional radiography device

The effective doses resulting from the Shimadzu FH‐21 HR radiographic device were 0.5 µSv for AP and 1.0 µSv for the LAT view (AP + LAT 1.5 µSv). The highest contributor to the effective dose was bone marrow (43%) followed by lymphatic nodes (28%), skin (17%), bone surface (7%), and muscles (4%).

### 3.B MSCT device

The effective dose obtained using Siemens Sensation Open MSCT device was 37.4 µSv. The major contributors to the effective dose were bone marrow (51%), lymphatic nodes (23%), skin (15%), bone surface (7%), and muscles (4%).

### 3.C CBCT devices

The effective dose acquired using NewTom 5G device (12 cm × 8 cm FOV) and “Standard Scan” setting was 2.0 µSv, and for the “HiRes” setting the effective dose was 6.7 µSv. The contributors to the effective dose for both dose settings were: bone marrow (50%), lymphatic nodes (29%), skin (11%), bone surface (6%), and muscles (4%). Using the 15 cm × 12 cm FOV with the “Standard Scan” setting resulted in an effective dose of 2.5 µSv. The contributors to the effective dose originated from bone marrow (50%), lymphatic nodes (28%), skin (12%), bone surface (6%), and muscle (4%). Using the 18 cm × 16 cm FOV, the NewTom 5G resulted in an effective dose of 2.1 µSv, and the effective dose contributions were bone marrow (48%), lymphatic nodes (26%), skin (15%), bone surface (7%), and muscle (5%).

The effective dose attained using Planmed Verity CBCT device was 2.6 µSv. The major contributors to the effective dose for both protocols were as follows: bone marrow (52%), lymphatic nodes (23%), skin (14%), bone surface (7%), and muscles (4%).

The effective doses, normalized effective dose (µSv/mAs), effective dose contributions, and dose comparison with the radiography dose (normalized to 1) for all protocols are presented in Table 4. The absorbed organ doses (mGy) and their dose comparisons with the average dose value of the radiography device are presented in Table 5.

**Table 5:** Absorbed organ doses (mGy) of elbow with radiography, MSCT and CBCT devices.

Dosim.	Organ	Shimadzu	Siemens	NewTom 5G				Planmed
		FH‐21 HR	Sensation	CBCT				Verity
		AP+LAT	Open	"Hi Res"	"Standard Scan"			CBCT
		Radiography	MSCT	12 × 8	12 × 8	15 × 12	18 × 16	13 × 16
1	Humerus	0.3	11.5	0.3	0.1	0.5	0.5	0.3
2	Humerus	0.5	17.1	2.9	0.9	0.8	1.0	1.3
3	Triceps brachii	0.6	18.8	1.5	0.5	1.1	1.1	1.4
4	Bicep brachii	1.1	21.6	4.9	1.3	1.1	1.3	1.4
5	Humerus	0.5	16.4	3.3	1.0	0.8	0.8	1.4
6	Cubital nodes/	0.9	18.5	4.2	1.3	1.5	1.2	1.3
7	Ulna	0.3	15.1	3.0	1.0	0.9	0.9	1.3
8	Ulna	0.4	14.9	2.6	0.7	1.2	0.5	1.0
9	Radius/Flexor c.	1.1	19.3	0.5	0.2	0.6	1.0	1.1
10	Ulna	0.4	13.3	0.3	0.1	0.9	0.5	0.7
11	Ulna	0.4	11.1	0.6	0.2	1.0	0.3	0.4
Average dose		0.6	16.1	2.2	0.7	1.0	0.8	1.1
Absorbed organ dose ratio^a^		1.0	28.4	3.9	1.2	1.7	1.5	1.9

^a^ Dose compared to the average absorbed organ dose of the radiographic device.

**Fig. 4:**
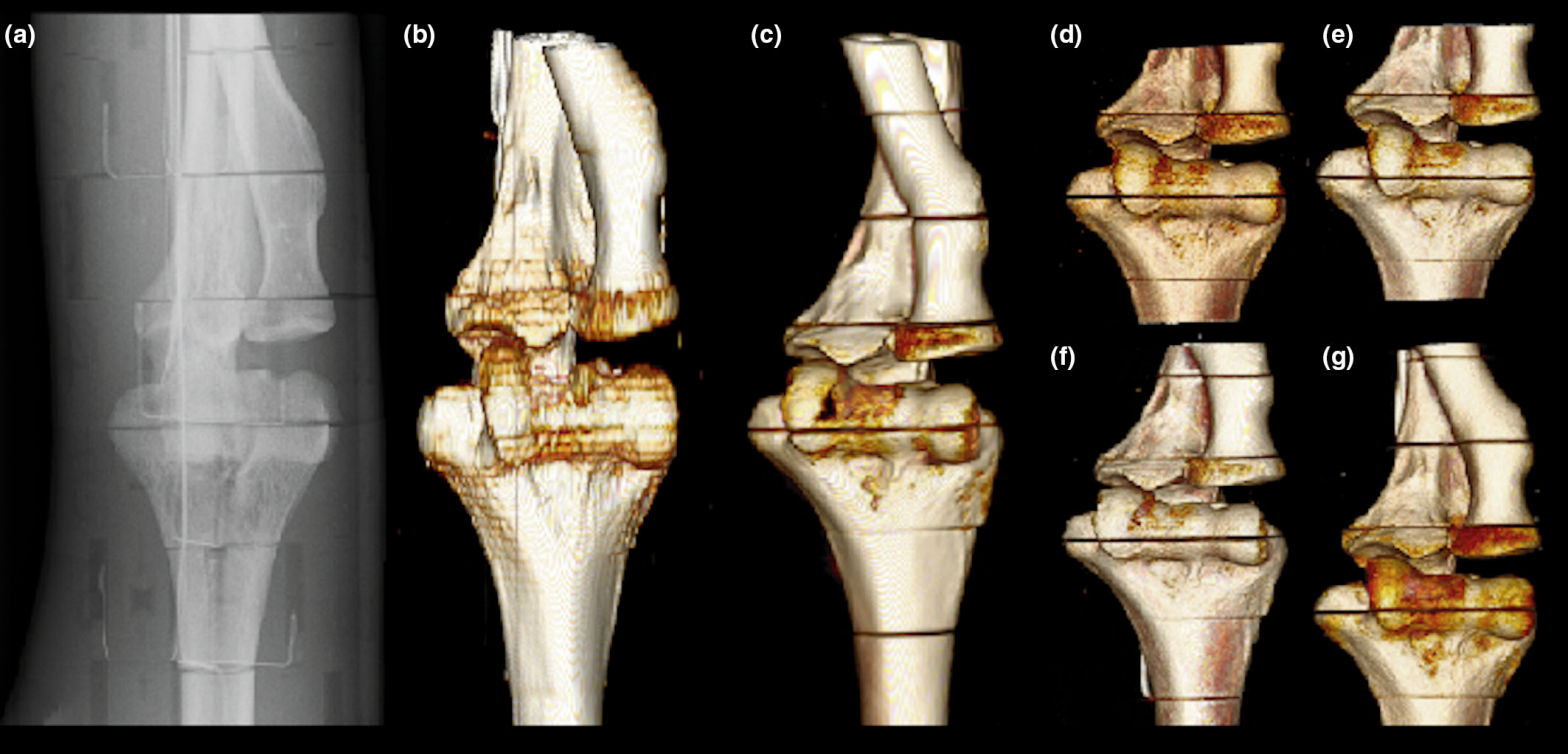
Anterior‐posterior elbow projections using the Shimadzu FH21‐HR radiography device (a), the Siemens Sensation Open MSCT device (b), the Planmed Verity CBCT device (c) and the NewTom 5G CBCT device HiRes 12 × 8 cm FOV (d), Standard Scan 12 × 8 cm (e), Standard Scan 15 × 12 cm FOV (f), Standard Scan 18 × 16 cm FOV (g).

The anterior‐posterior projections (cropped images) of the exposed volume using conventional radiographic, two CBCT, and MSCT devices are presented in Fig. 4.

### 3.D Uncertainty, effective and organ doses

The type A standard uncertainty of the absorbed dose for all protocols varied between 15% and 48% in the 11 dosimeters. The uncertainties of point dose measurements were calculated as weighted sum of variances and included the statistical measurement error of ten repeated measurements according to a previous study,^34^ dosimeter‐ and phantom position uncertainties, (10% and 10% respectively), x‐ray source variation (5%) and cable irradiation uncertainties (1%).^35^ The tissue dose uncertainty depended on the dosimeter uncertainty and the estimated fraction of irradiated *f_i_* (25%).

The Type B effective dose uncertainties (1SD, 2SD) were calculated as weighted sum of variances of bone marrow, bone surface, skin, and remainder tissues. The uncertainty (1SD and expanded 2SD) results were as follows: Shimadzu (14%, 28%), Siemens Sensation Open (12%, 24%), Planmed Verity (13%, 26%), NewTom 12 cm × 8 cm “HiRes” (14%, 29%) and for NewTom “Standard Scan”: 12 cm × 8 cm (15%, 30%), 15 cm × 12 cm (14%, 28%), 18 cm × 16 cm (13%, 26%).

## 4 DISCUSSION

In this paper, the absorbed and effective doses in the elbow region were evaluated on one radiographic device, two CBCT devices and one MSCT device. Two‐dimensional radiography typically consists of AP and LAT projections and is the most commonly used diagnostic imaging method in the elbow area. Therefore, the effective dose resulting from radiography projections was used as a benchmark for the dose comparison.

### 4.A Effective dose

The concept of effective dose was first introduced in 1977 to assess stochastic health effects of radiation in medicine.^36^ Since the introduction of effective dose, there has been an ongoing debate about its suitability for dose assessment due to the uncertainties in the conversion coefficients that are used to calculate the tissue weighting factors.^37^ Regardless of the drawbacks, effective dose is, however, the only means used to assess and compare the risk of health detriment and was therefore chosen for this study.^38^ However, according to Fisher et al.^39^ the individual assessment of potential detriment should be based on organ or tissue absorbed radiation dose. Therefore, the measured absorbed organ doses of each protocol were also included in this study for dose comparison. The effective dose ratios (Table 4) and the absorbed organ dose ratios of the corresponding protocol mean absorbed doses (Table 5) have an excellent correlation (0.9999).

#### 4.B.1 Effective dose comparison

The highest effective dose (37.4 µSv) was attained on the Siemens Sensation MSCT device. The effective dose was 25.4 times the effective dose measured on the 2D radiographic device (1.5 µSv). In contrast, the NewTom 5G CBCT effective dose was only 1.4 times the dose attained on the 2D radiographic device using the “Standard Scan” settings 12 cm × 8 cm FOV (2.0 µSv). Using the same FOV (12 × 8 cm) and the “HiRes” setting on the New Tom device, the effective dose was markedly higher (6.7 µSv) and was 4.6 times the dose of the radiographic device. When using the “Standard Scan” setting, the effective doses were 2.5 µSv for 15 cm × 12 cm FOV and 2.1 µSv for 18 cm × 16 cm FOV being 1.7 and 1.4 times the dose attained using the 2D radiographic device. The effective dose recorded on the Planmed Verity CBCT device (2.6 µSv) was 1.8 times the effective dose measured on the 2D radiographic device. When compared to the CBCT device, the effective dose attained on MSCT device was between 6 to 19 times the dose attained using the NewTom CBCT device and 14 times dose acquired on the Planmed Verity CBCT device.

The difference between MSCT effective dose and the average effective dose of the investigated CBCT protocols was 34.2 µSv. Although this dose difference may seem negligible, being equivalent to only 1% (4 days) of the yearly background radiation dose (3.1 mSv),^40^ it is still comparable to the effective dose resulting from two dental panoramic examinations.^41^ In comparison, the effective dose difference between the radiography (1.5 µSv) and the CBCT dose on average (3.2 µSv) was 1.7 µSv, which is comparable to 5 h of background radiation.

#### 4.B.2 CBCT protocol comparison

When comparing the CBCT devices the effective doses acquired using the NewTom 5G “Standard Scan” setting were between 4% and 22% lower (12 cm × 8 cm, 15 cm × 12 cm, and 18 cm × 16 cm FOV) than the effective dose measured on the Planmed Verity CBCT device (FOV 13 cm × 16 cm). These differences can be explained by the significantly lower tube‐current exposure–time product (mAs) values used in the NewTom 5G device (12 cm × 8 cm FOV: 3.5 mAs, 15 cm × 12 cm FOV: 2.6 mAs and 18 cm × 16 cm FOV: 2.4 mAs) when compared with the Planmed Verity 22.5 mAs value. However, when compared to the (3.5 mAs) tube current –exposure product of the “Standard Scan” the “HiRes” mode uses 7.9 times higher (27.7 mAs) current‐exposure product.

Since the dose is linearly dependent on the mAs‐value, the effective dose of the “HiRes” mode should also be 7.9 higher than that of the “Standard Scan” mode. Surprisingly, the effective dose of the “HiRes” mode resulted in only a 3.4 times higher value than the “Standard Scan” mode. This finding is in good agreement with previous studies.^20^, ^21^ Surprisingly, when using the larger FOVs on the NewTom 5G, lower mA values were recorded. This phenomenon could be caused by the “Safe Beam” technology that automatically adjusts the exposure current according to the patient’s anatomic density observed in the scout images. However, when using larger FOV the elbow shadows a smaller fraction of the detector area that subsequently causes underestimation of the patient attenuation and the use of smaller mA value.

It must be noted that both CBCT devices use inherently different exposure parameters and FOV settings. The NewTom 5G uses a constant 110 kVp tube voltage and does not offer a manual tube current adjustment. Furthermore, the NewTom 5G offers different FOV settings: 18 cm × 16 cm, 15 cm × 12 cm, 12 cm × 8 cm, 8 cm × 8 cm, 15 cm × 5 cm and 6 cm × 6 cm. The Planmed Verity CBCT device, on the other hand, offers adjustable mA‐ values ranging between 4.8 and 12 mA but offers only one (13 cm × 16 cm) FOV.

The low mAs value is an obvious exposure reduction benefit and subsequently results in a lower effective dose. However, the low mAs value could have a negative influence on the image quality since the contrast‐to‐noise‐ratio (CNR) of the image is inversely proportional to the mAs^0.5^.^42^ Furthermore, the CNR is known to be closely associated with the image quality.^43^, ^44^, ^45^

In a previous study, Biswas et al.^46^ quantified the effective dose from musculoskeletal CT imaging using a GE Lightspeed 16 Qx/I MSCT device (GE Medical Systems, Milwaukee, WI, USA). The effective dose was calculated according to a modified protocol derived from publication SR250 of the National Radiological Protection Board of the United Kingdom.^47^

The effective dose they recorded in the elbow area was 0.14 mSv. In our study, the MSCT effective dose was 37 µSv, being 26% of that observed by Biswas et al. The higher tube current used by Biswas et al. explains the difference in the dose. Another explaining factor for the differences in the effective doses are the dosimetric methods used. The physically realized RANDO phantom and mathematically modeled phantom are known to cause differences in the effective dose values.^34^

In an earlier study, Cross et al.^48^ investigated the effective dose in the elbow resulting from conventional radiographic diagnostic imaging. In their study, the entrance dose was measured and the effective dose calculated using Monte‐Carlo simulations. Their study resulted in a 0.003 mSv effective dose that was two times the dose attained in this study. Furthermore, in an earlier study, Huda et al.^49^ evaluated the effective dose in the elbow area using a radiographic device (Philips Classic C‐850 generator, Eureka ROT‐350 x‐ray tube). The effective dose assessment was based on the evaluation of the imparted energy to the elbow (66 kVp, 10 mAs). Their study resulted in a 0.69 µSv effective dose using one imaging projection. This result is in good agreement with the 1.5 µSv effective dose sum for two projections attained in this study.

One problem faced in the current study is that to date there is no general consensus on the quantity of red bone marrow in the arms. Cristy et al.^50^ reported that red bone marrow is generally not found in the arms. On the contrary, a recent study by Karampinos et al.^51^ reported that red bone marrow can be found in the ends of the long bones near the joints in healthy adults. Moreover, in a recent study, Machann et al.^34^ reported on red and yellow bone marrow distributions in young children and adults. More specifically, there is an age‐related change in the distribution of active marrow ranging from a high of as much as 5% in infancy and early childhood to as low as 0% in adults. Therefore, the 1% red bone marrow content used in this study elbow of the total RBM was chosen as this reasonably reflects maximum potential adult marrow content and is a conservative estimator for the risk calculation.

**Fig. 5:**
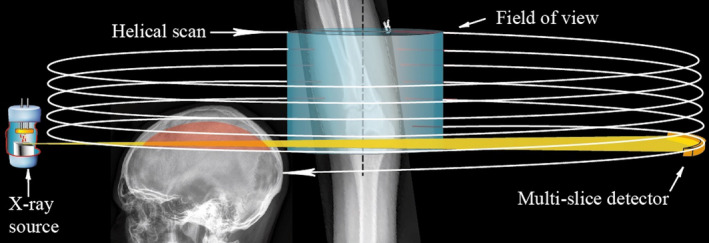
Exposing the elbow in an upright position using a CT device.

One difficulty when imaging elbow fractures using an MSCT device compared with a dedicated extremity CBCT device is the positioning of the elbow into the FOV without irradiating other body parts. If the arm would be placed adjacent to the patient’s body, the internal organs would become exposed by the radiation. This would increase the effective dose markedly and cause radiation beam hardening and respiratory motion artifacts from the torso. In an optimal positioning of the elbow into MSCT device the arm is raised straight above the patient’s head. This arm positioning, in turn, according to a study by Chapman et al., causes dose to the bone marrow in the calvaria and to the brain as the upper part of the head becomes irradiated by the primary x‐ray fan beam^10^ (Fig. 5.). In order to evaluate the possible effective dose to the skull during the elbow CT examination, the head effective dose assessment results of a previous study by Koivisto et al. were used.^41^ In their study, the FOV of a CBCT device was directed in different elevations of the skull and the effective dose was assessed using MOSFET dosimeter measurements and PCXMC Monte Carlo simulations respectively. Using 84 kVp and 145 mAs, the upper part of the skull (elevation coordinate Z = 95 cm) was estimated to increase the overall effective dose by 40 µSv.

Although the image quality was not assessed in this study, similar dose assessments (80–96 kVp and 3.8–12 mA) to those in this study were undertaken by Huang et al. on an identical Planmed Verity CBCT device (Table 6). Furthermore, image quality assessments were also performed. The aim of their study was to evaluate whether it is justified to use CBCT technology instead of MDCT for extremity imaging in terms of image quality and radiation dose. The results of their study demonstrated that the Planmed Verity CBCT images yielded more diagnostic information than radiographs in 23 out of 51 cases and more diagnostic information than MDCT in one of seven cases. More specifically, they reported that CBCT images showed fracture lines that were either not or barely detectable on radiographs or MDCT device. Moreover, they concluded that the Planmed Verity CBCT device uses less imaging time than conventional radiographs and generates lower radiation doses than the Philips and GE MDCT scanners used in their study. It should be noted, however, that the MDCT devices used in the study by Huang et al. were not identical to the one used in the current study. However, the MDCT kVp values were identical to those used in our study and only minor differences in the mAs values were reported. Similar image quality versus effective dose findings were reported by Faccioli et al.^12^ Furthermore, previous studies by Suojärvi et al. and De Smet et al reported that CBCT technology was significantly better at detecting small bone and joint fractures when compared to radiographic devices. However, it must be noted that the increased detection rate of fractures resulted in higher radiation doses.^52^, ^53^, ^54^

**Table 6:** Effective doses (µSv) of the Planmed Verity CBCT using the standard dose kVp and mA exposure values.

	3.8 mA	7.5 mA	12 mA
80 kVp	1.15	2.27	3.63
84 kVp	1.34	2.65	4.24
88 kVp	1.63	3.21	5.14
90 kVp	1.74	3.44	5.50
92 kVp	1.96	3.88	6.20
96 kVp	2.30	4.54	7.26

Future studies are needed to investigate the MSCT and CBCT iterative reconstruction possibilities to reduce the effective dose while maintaining good diagnostic image quality.

## 5 CONCLUSIONS

When compared with the conventional radiographic device, the standard MSCT protocol resulted in a 25‐fold increase in effective dose. The standard elbow protocols on the NewTom 5G and Planmed Verity CBCT devices resulted in a 0.7‐ to 2.4‐fold increase in effective dose, respectively. The two CBCT devices offered 3D images of the elbow at a significantly lower dose than the MSCT device.

## AUTHOR CONTRIBUTION STATEMENT

Koivisto, J, Design of the effective dose measurement concept, absorbed organ dose acquisitions, calculation of effective doses. Writing the paper. Van Eijnatten, M, Analysis and interpretation of the results. Critical revising the article for intellectual content. Kiljunen, T, Defining the examined field of view in the elbow phantom, performing the uncertainty analysis and calculations. Ludlow, J, Assessment of tissue weighing factors and fractions irradiated in the elbow phantom, verification of the effective dose calculations. Revising the manuscript. Wolff, J, Design of the effective dose measurement concept, verifying the dosimeter anatomical locations in the elbow. Critical revising and final approval of the version to be published.

## CONFLICT OF INTEREST

No conflict of interest.

## Data Availability

Data available on request from the authors.
